# Length-dependent degradation of single-stranded 3' ends by the Werner syndrome protein (WRN): implications for spatial orientation and coordinated 3' to 5' movement of its ATPase/helicase and exonuclease domains

**DOI:** 10.1186/1471-2199-7-6

**Published:** 2006-02-17

**Authors:** Amrita Machwe, Liren Xiao, David K Orren

**Affiliations:** 1 Graduate Center for Toxicology, College of Medicine, 800 Rose Street, University of Kentucky, Lexington, KY 40536-0305, USA

## Abstract

**Background:**

The cancer-prone and accelerated aging disease Werner syndrome is caused by loss of function of the *WRN *gene product that possesses ATPase, 3' to 5' helicase and 3' to 5' exonuclease activities. Although WRN has been most prominently suggested to function in telomere maintenance, resolution of replication blockage and/or recombinational repair, its exact role in DNA metabolism remains unclear. WRN is the only human RecQ family member to possess both helicase and exonuclease activity, but the mechanistic relationship between these activities is unknown. In this study, model single-stranded and 3' overhang DNA substrates of varying length and structure were used to examine potential coordination between the ATPase/helicase and exonuclease activities of WRN.

**Results:**

Our results show that WRN can not only bind to but also catalyze the 3' to 5' degradation of single-stranded and 3' overhang DNA substrates, structures that were previously thought to be refractory to WRN exonuclease activity. The length of the single-stranded regions in these structures is a critical parameter in determining both the binding affinity and the level of exonuclease activity of WRN. Most importantly, specific nucleotide cofactors dramatically stimulate WRN exonuclease activity on these substrates, with conditions that permit ATP hydrolysis not only resulting in enhanced exonuclease activity but also altering its length dependence on these structures. Parallel experiments show that a deletion mutant containing only the WRN exonuclease domain lacks both this DNA length and nucleotide cofactor dependence, demonstrating that the interaction of the ATPase/helicase domain of WRN with the DNA substrate has a profound influence on exonuclease activity.

**Conclusion:**

Our results indicate that, under conditions that permit ATP hydrolysis, there is a dynamic and cooperative relationship between the distinct ATPase/helicase and exonuclease domains of WRN with regard to their orientation on DNA. Based on these results, models are proposed for the coordinated, unidirectional 3' to 5' movement of the helicase and exonuclease domains of WRN on DNA that should be informative for elucidating its function in genome maintenance.

## Background

Werner syndrome (WS) is a rare autosomal recessive disease displaying accelerated development of many characteristics associated with normal aging including graying and loss of hair, cataracts, osteoporosis, atherosclerosis, hypertension and increased frequencies of diabetes mellitus type II and specific types of cancer (reviewed in [1-6]). Individuals with WS normally die prior to age 50 from either heart disease or cancer. This segmental progeroid condition appears to be due to increased genomic instability caused by the loss of function of a single gene product, WRN. Thus, WS and the WRN protein have been utilized as models to understand potential relationships between genetic change over time and development of certain aging characteristics.

WRN belongs to the RecQ family of helicases [7] that has five human members including the BLM and RecQ4 proteins that are defective in the cancer-prone Bloom and Rothmund-Thomson syndromes, respectively [8,9]. In general, a cellular deficiency in a RecQ family member results in an illegitimate recombination phenotype, suggesting key roles for RecQ helicases in minimizing large-scale genome rearrangements. Although their exact metabolic functions remain unclear, commonly proposed roles for RecQ helicases involve participation in the disruption of aberrant recombination and in the resolution of replication blockage by fork regression or recombinational repair pathways [10-12]. WRN-deficient cells show a genomic instability phenotype typified by increased chromosomal abnormalities (translocations, deletions, and insertions) and abnormal telomere dynamics [13-15], again consistent with an important role for WRN in suppressing genome change. WRN-deficient primary fibroblasts also undergo a rapid premature cellular senescence that may be due to loss of a specific telomeric function [16,17]. Importantly, accumulation of senescent cells and/or loss of cells by apoptosis have been proposed as putative mechanisms at work in development of aging phenotypes not only in WS but also during normal aging [18,19].

The WRN protein possesses a central ATPase/helicase domain composed of defined sequence motifs that are highly homologous within RecQ family members. In all RecQ helicases characterized thus far, this domain is essential for both (single-stranded) DNA-dependent ATPase and nucleic acid unwinding activities [10,12]. WRN and other RecQ members utilize ATP hydrolysis to unwind DNA with a 3' → 5' directionality, defined with respect to the strand to which the helicase is bound. Consistent with this polarity, a single-stranded region 3' to the duplex to be unwound (i.e., a 3' overhang) permits WRN helicase activity on partial duplex substrates [20-22]. Although a region with single-stranded character is not an absolute requirement for WRN-mediated unwinding, biochemical studies thus far are consistent with a mechanism in which WRN can preferentially bind to junctions between a 3' single-stranded region and duplex DNA, and unwind the duplex by moving in a 3' → 5' direction [20,22].

The WRN protein also has an N-terminal nuclease domain that is not present in other human RecQ members [23]. Biochemical characterization indicates that this N-terminal domain functions as an exonuclease, the polarity of which is also 3' → 5' [24-26]. For *in vitro *studies, the catalytic functions of the exonuclease and helicase domains can be isolated by making either key amino acid substitutions in each domain or deletion mutants lacking one region or the other [24,27,28]. Although this indicates that the exonuclease and helicase domains are spatially separated within the three-dimensional structure of WRN and can act independently *in vitro*, it is unclear how these domains act with respect to one another on relevant DNA structures *in vivo*. Clarification of the relationship between the exonuclease and ATPase/helicase activities may be imperative for understanding the role of WRN in DNA metabolism, as the loss of their combined function may be involved in generating the unique WS phenotype.

Experiments using model DNA substrates of varying length and structure can be valuable for investigating this potential coordination between the exonuclease and ATPase/helicase domains of WRN. Early experiments on relatively short DNA substrates suggested that WRN exonuclease has limited substrate specificity, preferring partial duplexes with a recessed 3' end structure [25,29]. Here, our experiments with significantly longer DNA substrates (single-stranded oligomers and 3' overhangs) reveal a much wider substrate specificity for the 3' → 5' exonuclease activity of WRN. Specifically, this study demonstrates extensive WRN-mediated degradation of both single-stranded DNA and duplexes with 3' overhangs as well as a profound effect of the length of the single-stranded region on exonuclease activity. Importantly, our experiments also show that ATP binding and hydrolysis stimulate WRN exonuclease activity in a manner influenced greatly by substrate length and structure. Comparative experiments using a deletion mutant containing only the N-terminal exonuclease domain do not show an effect of nucleotide cofactors or any dependence on the length of single-stranded DNA in our substrates. Altogether, these results are novel and particularly noteworthy in that they emphasize a direct relationship between WRN's exonuclease and ATPase/helicase domains that is specifically influenced by DNA structure and length, and indicate that the ATPase/helicase and exonuclease domains engage in concerted 3' → 5' movement and catalytic function on appropriate and physiologically relevant DNA structures.

## Results and discussion

### WRN exonuclease activity on single-stranded DNA is length dependent

**Figure 1:**
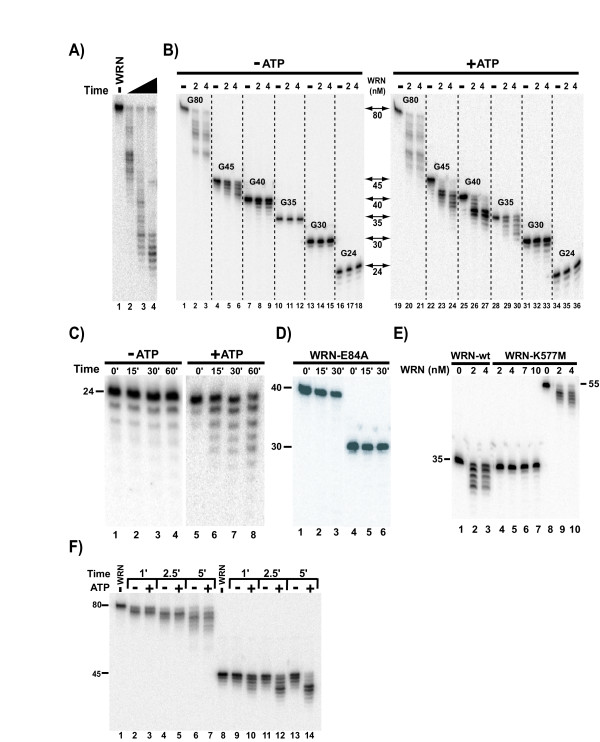
**WRN exonuclease activity on single-stranded DNA is dependent on length and ATP hydrolysis. A) **Standard exonuclease assays were performed on single-stranded, labeled G80 (0.1 nM) for 15, 30 or 60 mins at 37°C with WRN (10.8 nM) and ATP (1 mM). **B) **Similarly, exonuclease assays were also performed for 15 min at 37°C on labeled oligomers of different lengths (G80, G45, G40, G35, G30, G24, 0.1 nM each) with WRN (2 or 4 nM) either without (*left panel*) or with 1 mM ATP (*right panel*). **C) **Reactions containing WRN (10.8 nM), labeled G24 (0.1 nM) and without (*lanes 1–4*) or with (*lanes 5–8*) 1 mM ATP were incubated for 15, 30 or 60 min and assayed for exonuclease activity. **D) **Exonuclease-deficient WRN-E84A (22 nM) was incubated with 0.1 nM of labeled G40 (*lanes 1–3*) or G30 (*lanes 4–6*) and 1 mM ATP for 0, 15 or 30 min. **E) **As indicated, either WRN (2–4 nM) or ATPase/helicase-deficient WRN-K577M (2–10 nM) was incubated with G35 or C55 (0.1 nM each) for 15 min at 37°C in the presence of 1 mM ATP. **F) **WRN (3.4 nM) was incubated with G80 or G45 (0.1 nM each) without or with 1 mM ATP as indicated for 1, 2.5 or 5 min. For each experiment above, reactions were stopped and DNA products were analyzed as described for the exonuclease assay in Methods; the positions of migration and lengths of the original substrates (in nt) are indicated between or at left of the panels above.

In previous studies, we have observed that WRN acts very efficiently on long DNA substrates (> 50 bp/nt) that contained at least partial single-stranded character [30,31]. Accordingly, wild type WRN also showed extensive stepwise degradation of an 80 nt single-stranded oligomer over 1 hr (Fig. 1A), with the same 3' → 5' polarity observed on various duplex substrates [24-26,32]. These results contrast with earlier reports that did not reveal any significant 3' → 5' exonuclease activity of WRN on single-stranded oligomers of shorter length--i.e., ≤ 24 nt [25,29]. Thus, the exonuclease activity of WRN on linear single-stranded DNA or other structures might be influenced by substrate length. To investigate this possibility, individual single-stranded oligomers ranging in lengths from 24 to 80 nt [G24, G30, G35, G40, G45 and G80, each sharing a minimum of 24 nt at their 3' ends (see Table 1)] were incubated with WRN for 15 min without ATP and DNA products were separated by denaturing PAGE. WRN readily attacks the 3' end of the 80-mer, removing one or more nucleotides from most molecules of the original substrate, with some individual molecules being degraded by as much as 20 nt (Fig. 1B, *lanes 1–3*). When the substrate length is 45 nt, exonuclease activity is still observed, but the extent of inward degradation is limited to < 5 nt and much of the original 45-mer remains undigested (Fig. 1B, *lanes 4–6*). With a 40-mer, only a few nucleotides are removed from a minority of the substrate, with most of the original substrate still intact (Fig. 1B, *lanes 7–9*). When even shorter oligomers of 35, 30 or 24 nt were used (Fig. 1B, *lanes 10–18*), no change was observed, indicating that WRN exonuclease does not degrade single-stranded substrates of ≤ 35 nt, at least in the absence of ATP. These results demonstrate that WRN exonuclease activity on single-stranded DNA is indeed length-dependent.

In addition to its exonuclease function, WRN also binds and hydrolyzes ATP to drive DNA unwinding. Although ATP is not required for WRN exonuclease activity on some DNA structures, we considered the possibility that ATP might influence this activity on single-stranded substrates. Therefore, exonuclease assays with various length oligomers were essentially repeated, except the reactions contained ATP. As in reactions without ATP, WRN degraded the 80-mer extensively in the presence of ATP (Fig. 1B, *lanes 19–21*). However, addition of ATP markedly improved WRN-mediated degradation of the 45, 40, 35, and 30 nt oligomers. In sharp contrast to parallel reactions without ATP, there is extensive degradation of the 45- and 40-mers in reactions containing ATP (Fig. 1B, *compare lanes 4–9 with 22–27*), with these digestion patterns revealing both a near complete absence of the original substrate and a much greater extent of inward degradation (most molecules degraded by ≥ 4 nt). The 35 nt oligomer that was not detectably digested by WRN in the absence of ATP is now significantly degraded in its presence (Fig. 1B, *compare lanes 10–12 with 28–30*). Specifically, after 15 min approximately half of the original substrate was digested, with as many as 5 nt being removed. Similarly, the 30-mer that was not digested by WRN in the absence of ATP was modestly degraded (by up to 3 nt) in its presence (Fig. 1B, *compare lanes 13–15 with 31–33*). When the oligomer length was 24 nt, no change was observed when the same amount of WRN was added in the presence or absence of ATP (Fig. 1B, *compare lanes 16–18 with lanes 34–36*). However, by significantly increasing both incubation time and WRN concentration, limited digestion of even the 24-mer was observed, but only in the presence of ATP (Fig. 1C). We wanted to confirm that this exonuclease activity was specific, since this is the first demonstration that WRN alone degrades single-stranded DNA. Thus, experiments with the various length oligomers were performed using WRN-E84A that contains an amino acid substitution that eliminates WRN exonuclease activity [24]. In reactions containing WRN-E84A and ATP, no 3' → 5' degradation of the 30- or 40-mer (Fig. 1D) or other single-stranded substrates of shorter or longer length either in the presence or absence of ATP was detected (data not shown). Similarly, single-stranded oligomers were incubated with the ATPase- and helicase-deficient mutant WRN-K577M to determine whether the ATP-dependent stimulation of exonuclease activity was specifically due to the ATPase/helicase domain of WRN. In contrast to wild type protein, WRN-K577M cannot act on the 35-mer in the presence of ATP; however, it can degrade a longer (55 nt) oligomer in a limited manner (Fig. 1E), similar to wild type WRN without ATP. Thus, our results with mutant WRN proteins indicate that the 3' → 5' digestion of single-stranded oligomers and its stimulation by ATP are indeed due to the inherent exonuclease and ATPase/helicase activities of WRN.

This ATP-dependent enhancement of exonuclease activity on shorter (≤ 45 nt) oligomers suggested that the central ATPase/helicase domain of WRN was influencing its N-terminal exonuclease function. Two possibilities were considered to explain this effect: 1) ATP binding or hydrolysis converts WRN into a conformation that makes it a more efficient or processive exonuclease that is independent of DNA structure and length, or 2) ATP binding or hydrolysis affects the dynamics of WRN's interaction with DNA that also influences exonuclease activity. Since the former would be independent of substrate length while the latter might not, the rates of degradation by WRN exonuclease were compared in the presence and absence of ATP on the 80 and 45 nt oligomers. A typical experiment (Fig. 1F) not only confirmed the effects of ATP and substrate length observed above, but also showed that, while the rates and patterns of digestion of the 80-mer are essentially identical without or with ATP, both the rate and extent of digestion of the 45-mer is much greater in the presence of ATP than in its absence. Since varying MgCl_2 _concentrations between 2–6 mM in the presence or absence of ATP (1 mM) did not significantly alter WRN exonuclease activity (data not shown), differences in digestion rates or patterns were not due to a non-specific effect of the free Mg^+2 ^concentration. Thus, these experiments demonstrate that ATP enhances exonuclease digestion only when oligomer length becomes limiting, and indicate that the ATPase/helicase domain influences WRN interactions with DNA that, in turn, affect its exonuclease function.

### Binding of WRN to single-stranded DNA

**Figure 2:**
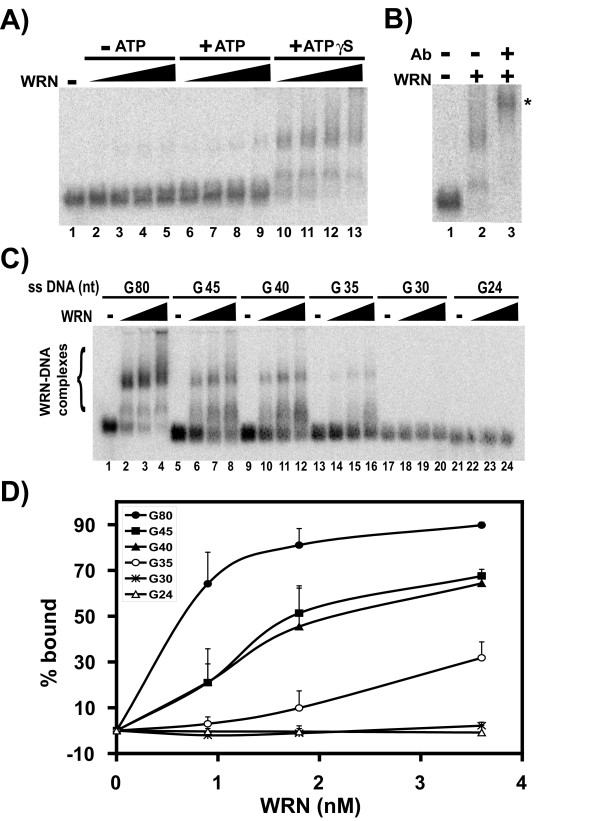
**WRN binding to single-stranded DNA is length-dependent. **For only these EMSA experiments, oligomers contained a 3'-PO_4 _group to block the exonuclease activity of wild type WRN. **A) **Wild type WRN (0.6, 1.2, 2.4, or 4.8 nM) was incubated with G80 (0.025 nM) in the presence or absence of ATP or ATPγS (1 mM), and free DNA and DNA-protein complexes were separated using EMSA as described in Methods. **B) **DNA-protein complexes formed by incubation of G80 (0.025 nM) with WRN (1.8 nM) in ATPγS were subsequently incubated with or without anti-WRN antibody as indicated and analyzed as in *A*. The slower mobility (supershift) of the protein-DNA complexes (*denoted by asterisk*) mediated by the anti-WRN antibody (*lane 3*) indicates that the complexes formed contain WRN. **C) **G80, G45, G40, G35, G30, or G24 (0.025 nM) was incubated without or with WRN (0.9, 1.8, or 3.6 nM) in ATPγS and products were analyzed as in *A*. **D) **From EMSA experiments as in *C*, quantitation and graphical representation of WRN binding to the G80 (●), G45 (■), G40 (▲), G35 (○), G30 (×), and G24 (△) oligomers. Each data point is the mean of three independent experiments, with the exception of G24 (two experiments).

The results above indicated that WRN exonuclease degrades single-stranded oligomers in a manner dependent on their length and influenced by nucleotide cofactors. One possible explanation for these results is that the length of single-stranded DNA determines WRN binding affinity and thus its ability to degrade a substrate. In order to assess WRN binding affinity for single-stranded oligomers, electrophoretic mobility shift assays (EMSA) were performed. Initial experiments to determine conditions for EMSA indicated optimal formation of WRN-DNA (80-mer) complexes when binding reactions contained the poorly hydrolyzable analog ATPγS as compared to reactions with or without ATP (Fig. 2A). Higher WRN concentrations were needed to observe significant amounts of protein-DNA complexes when reactions either contained ATP or lacked nucleotide cofactors altogether (data not shown). Importantly, these shifted bands represent WRN-DNA complexes as they were supershifted by a WRN-specific antibody (Fig. 2B). These results indicate that the ATP-bound conformation of WRN forms the most stable complexes with single-stranded DNA.

To determine whether the apparent length dependence of WRN exonuclease activity was related to DNA binding capability, EMSA experiments were performed with oligomers of different length. These oligomers were identical to those in Fig. 1B, except that they were modified at their 3' ends specifically to block WRN exonuclease activity (see Methods: DNA substrates). The results of these experiments (Figs. 2C and 2D) clearly show that binding of WRN to single-stranded DNA is also influenced by its length. WRN binds most avidly to the 80-mer, with lower affinity to the 45- and 40-mers, and very weakly to the 35-mer. Under these conditions, no binding of WRN to the 30 and 24 nt oligomers is detected (Fig. 2C and 2D). Similarly, WRN binding strength was modulated by oligomer length even when these assays were performed without or with ATP instead of ATPγS (data not shown). If these binding assays are compared with our exonuclease results (see Fig. 1B), it certainly appears that oligomer binding by WRN correlates favorably to its degradation. Specifically, on the 24-and 30-mers where no binding is detected, no degradation is observed in the absence of ATP and very limited degradation in the presence of ATP. As oligomer length increases, the binding affinity of WRN increases as does the amount of 3' → 5' exonuclease activity. Importantly, this correlation is not exact and nucleotide cofactors play a key role. For example, even though there is modest binding of the 35-mer by WRN, no degradation is observed unless ATP is present. In the absence of ATP, the initial attack and subsequent degradation by WRN exonuclease require a longer oligomer length than needed for DNA binding; the presence of ATP allows significant degradation of oligomers of ≥ 35 nt and even limited degradation of 24- and 30-mers to which binding is not observed under our EMSA conditions. In summary, stable WRN binding positively correlates with the ability of the exonuclease domain to access and act on a single-stranded substrate, but a more dynamic (but less stable) interaction driven by ATP hydrolysis appears necessary for digestion of short (≤ 45 nt) oligomers.

### Processivity of WRN exonuclease on single-stranded DNA

**Figure 3:**
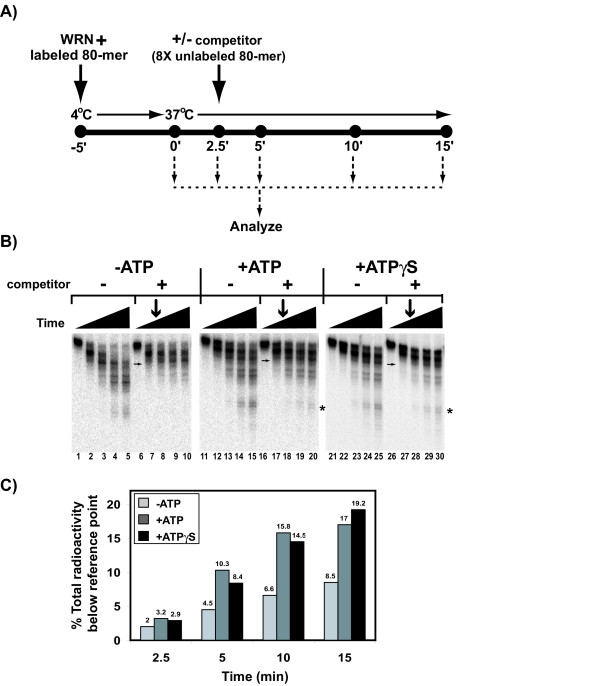
**The processivity of WRN exonuclease is enhanced by ATP binding and hydrolysis. A) **Experimental design to examine, under various conditions, processivity of WRN exonuclease activity on single-stranded substrates by addition of excess competitor single-stranded DNA during the reaction. In duplicate samples, labeled, single-stranded 80-mer is preincubated with WRN at 4°C for 5 min, then enzymatic reactions are initiated at 37°C. After 2.5 min, an excess of competitor DNA (unlabeled 80-mer) was added to one reaction while the other remained unchanged. For each sample, aliquots were removed for analysis just prior to the 37°C incubation (0 min), at the time of addition of the competitor (2.5 min), and at 5, 10, and 15 min into the 37°C incubation. **B) **As described above, reactions containing WRN (1.2 nM) and G80 (0.25 nM) without ATP (*left panel*), with 1 mM ATP (*middle panel*), or with 1 mM ATPγS (*right panel*) conditions were preincubated at 4°C then incubated at 37°C and, where indicated, an eightfold excess of unlabeled G80 (2 nM) was added 2.5 min into the 37°C incubation. Aliquots were removed after 0, 2.5, 5, 10, and 15 min of 37°C incubation for analysis of exonuclease activity as described in Methods. Vertical arrows indicate the timing of addition of excess competitor in the relevant reactions; asterisks indicate relatively short fragments appearing in reactions containing competitor as well as either ATP or ATPγS, but not in comparable reactions minus ATP. Horizontal arrows indicate the position of the reference point used in partitioning of long and short fragments for quantitation (*presented in C*) of the relative amounts of inward degradation under different conditions. **C) **For the reactions in *B *in which the unlabeled G80 competitor was added at 2.5 min, the percentage of radioactivity present below the reference point (*defined by horizontal arrows in B*) with respect to the total radioactivity in the reaction was quantitated. The bar graph directly compares the percentage of radioactivity below the reference point for the different reaction conditions (-ATP, +ATP, +ATPγS) at the individual time points; the exact percentage appears above its respective bar.

The ATP-stimulated degradation of single-stranded oligomers by WRN suggested possible cooperation between its exonuclease and ATPase/helicase domains. Research on many helicases has demonstrated that ATPase activity drives movement of the enzyme along single-stranded DNA or one strand of the duplex being unwound [33,34]. For WRN, the directionality of movement along single-stranded DNA is 3' → 5', the same as the polarity of exonuclease activity. Hypothetically, movement of the helicase domain along single-stranded DNA might facilitate digestion (or processivity) by the exonuclease domain being pulled along behind. Thus, experiments were designed to investigate the effect of nucleotide cofactors on the extent of inward digestion by WRN exonuclease on a single-stranded oligomer. In reactions without or with ATP or ATPγS, radiolabeled 80-mer was pre-incubated with WRN at 4°C to facilitate substrate binding, then the enzymatic reaction was initiated at 37°C and after 2.5 min, the reactions were either allowed to continue undisturbed or challenged with excess unlabeled 80-mer (Fig. 3A). Subsequently, degradation of labeled substrate at various time points without or with competitor was analyzed. Importantly, when a significant excess of unlabeled 80-mer is included, subsequent measurements of exonuclease activity represent degradation of the molecules of labeled substrate already bound by WRN – i.e., the competitor inhibits rebinding of WRN and re-initiation of degradation on labeled substrate molecules. Thus, a drastic reduction in the extent of inward degradation after the introduction of competitor at 2.5 min indicates transfer of WRN from the labeled substrate to the excess unlabeled oligomer and suggests a low processivity or mainly distributive mechanism of digestion. In contrast, if addition of competitor does not inhibit inward degradation, it suggests that WRN remains bound to the labeled oligomer and continues degradation of that molecule – i.e., a more processive mechanism of action.

The results of representative processivity experiments using an eightfold excess of unlabeled 80-mer in the absence or presence of ATP or ATPγS are shown in Fig. 3B. Under each condition, the amount of undigested labeled substrate remaining was only marginally reduced after addition of the competitor, confirming that the competitor soaked up almost all WRN that was either not bound to or released from the labeled substrate. When the digestion patterns for reactions with and without competitor are directly compared, the competitor did decrease the overall amount of WRN degradation under each condition. However, the degree of inhibition by competitor varied with different reaction conditions. Specifically, in reactions containing competitor, the amount of inward degradation was more extensive in ATP or ATPγS than without nucleotide cofactor (Fig. 3B, *compare lanes 7–10 with lanes 17–20 and lanes 27–30*). This greater extent of inward digestion in the presence of competitor under ATP or ATPγS conditions was demonstrated by the appearance of relatively short fragments (*indicated with asterisks*) that are not visible in comparable reactions without ATP. Since relative processivity is indicated by further inward digestion subsequent to addition of competitor, the distribution of radioactivity in each lane was measured with respect to a specific reference point in the digestion patterns for the 2.5 min time points (Fig. 3B, *indicated by horizontal arrows*), at which time the vast majority (> 95%) of signal was present in longer fragments (*above the arrow*). Thus, the amount of radioactivity present in shorter fragments (*below the arrow*) at time points beyond 2.5 min is a measure of the amount of inward degradation that occurs on those molecules already bound by WRN after addition of competitor. This analysis (Fig. 3C) shows that, regardless of the presence or absence of nucleotide cofactor, the amount of radioactivity present in fragments shorter than this reference point was minimal (< 3.2%) at the time (2.5 min) competitor was added. In reactions without ATP, the increase in the amount of shorter fragments with time was very modest, reaching only 8.5% of the total radioactivity in the reaction after 15 min. In reactions containing either ATP or ATPγS, radioactivity present in shorter fragments was 2–3 times greater at the 5, 10, and 15 min time points (i.e., after adding competitor) than in reactions without ATP. Specifically, these experiments showed that WRN, during a single round of binding, digested a single-stranded substrate significantly better under conditions where ATP binding and/or hydrolysis were permitted than when ATP was absent. In other words, WRN exonuclease activity is more "processive" when ATP is bound or hydrolyzed and more "distributive" in the absence of ATP. Notably, the extent of inward digestion in the presence of ATPγS may reflect a highly stable mode of binding to single-stranded DNA, as evidenced by EMSA (Fig. 2A).

### WRN exonuclease activity on 3' overhang substrates is influenced by overhang length

**Figure 4:**
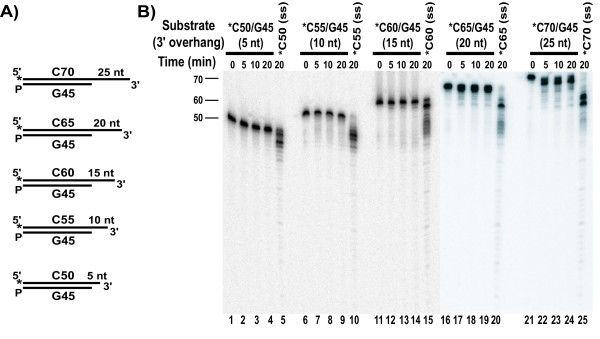
**WRN exonuclease can digest 3' overhangs of sufficient length. A) **Structures of the 3' overhang substrates. Unlabeled G45 oligomer was annealed to labeled C70, C65, C60, C55, or C50 oligomer to create 45 bp substrates with single-stranded 3' overhangs of 5–25 nt as indicated. The positions of radiolabels are depicted by asterisks; "P" represents the phosphate moiety added to the 3' end of the unlabeled G45 strand to prevent WRN degradation. **B) **Wild type WRN (2.7 nM) was incubated without ATP for the indicated times with the 5, 10, 15, 20, or 25 nt 3' overhang substrate (0.25 nM) or their single-stranded C50, C55, C60, C65, or C70 building blocks (0.25 nM) and assayed for exonuclease activity as described in Methods.

**Figure 5:**
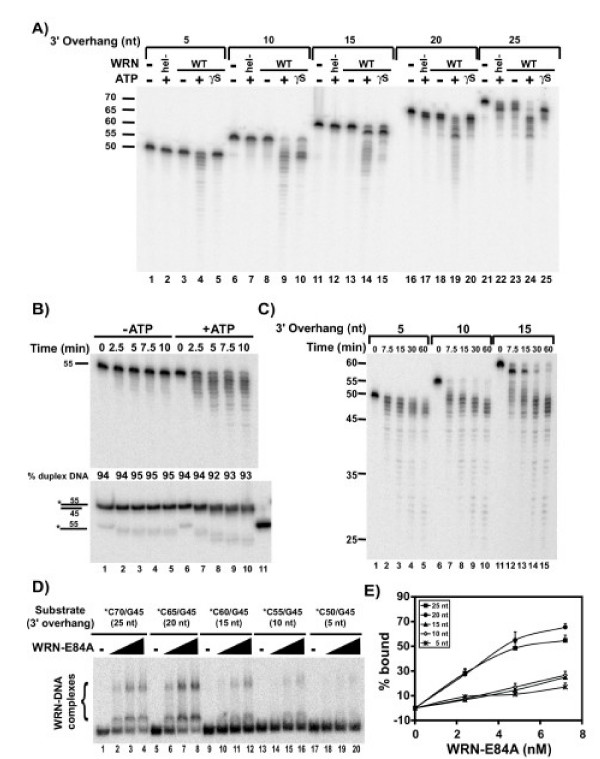
**ATP-dependent enhancement of WRN exonuclease on overhang substrates. A) **The 5, 10, 15, 20, or 25 nt 3' overhang substrate (0.25 nM) was incubated for 10 min at 37°C without enzyme, with helicase-deficient WRN-K577M (hel-, 3 nM), or with wild type WRN (WT, 2.7 nM) in the presence or absence of ATP or ATPγS (1 mM) as indicated. The initial lengths of the labeled strands for each substrate are indicated at left. **B) **Reactions containing the 10 nt 3' overhang C55/G45 substrate (0.25 nM) and wild type WRN (2.7 nM) were incubated without or with ATP (1 mM) as indicated at 37°C. At 0, 2.5, 5, 7.5, and 10 min, two aliquots were removed from each reaction; one was analyzed for exonuclease activity by denaturing PAGE (*top*) while the other was run on non-denaturing PAGE (*bottom*) to determine whether WRN was unwinding the substrate. The single-stranded, labeled C55 oligomer was loaded as a marker on the non-denaturing gel (*lane 11*) and the positions of undegraded 3' overhang substrate and single-stranded 55-mer are indicated at left. The extensive degradation observed under +ATP conditions (*top*) is almost exclusively occurring on the overhang substrate while still in duplex form, as indicated by its slightly faster migration with time (*bottom, lanes 7–10*). The small amount of signal migrating faster than the single-stranded marker in both the – and +ATP reactions is primarily due to degradation of the small amount of single-stranded component present in the original substrate preparation (*bottom, see lanes 1 and 6*). **C) **Reactions containing the 5, 10, or 15 nt overhang substrate (0.05 nM) were incubated 37°C with WRN (3.5 nM) in the presence of ATP (1 mM). Aliquots were removed at the indicated times and analyzed for exonuclease activity. Nucleotide size markers are indicated at left. **D) **As described in Methods, EMSA was performed with reactions containing ATPγS, WRN-E84A (2.4, 4.8, or 7.2 nM), and 5, 10, 15, 20, or 25 nt overhang substrate (0.025 nM) and as indicated. **E) **From EMSA experiments as in *D*, quantitation and graphical representation of WRN binding to the 25 (■), 20 (●),15 (▲),10 (◇), and 5 (×) nt overhangs. Each data point is the mean of two independent experiments.

Early characterization of the 3' → 5' exonuclease activity of WRN on partial duplex substrates suggested that the preferred end structure was a recessed 3' end, and that WRN alone was unable to digest substrates with 3' overhangs. However, these experiments used relatively short DNA substrates with overhangs of ≤ 13 nt [25,29]. Our results above suggested that degradation of 3' overhangs might be achieved if a sufficient length of single-stranded DNA was available. In order to investigate this possibility, a series of 45 bp substrates were constructed with 3' overhangs ranging from 5 to 25 nt (Fig. 4A). The ability of WRN exonuclease to degrade these overhang substrates over time was examined initially in reactions without ATP to prevent substrate unwinding, with a representative experiment depicted in Fig. 4B. Under these conditions, WRN clearly initiated degradation on a 25 nt 3' overhang, removing at least one nucleotide from most of the substrate molecules and digesting several nucleotides inward on some molecules (Fig. 4B, *lanes 21–24*). With shorter overhangs, exonuclease activity was much less pronounced or absent. Specifically, on the 20 nt overhang (Fig. 4B, *lanes 16–19*), WRN primarily removed only one nucleotide from the 3' end (see also Fig. 5A, *lane 18*, for better resolution of 64- and 65-mers) with very limited further inward degradation. On the 15 nt overhang, a single nucleotide was removed on only a very small percentage of substrate molecules (Fig. 4B, *lanes 11–14*). The 5 and 10 nt overhangs were not degraded significantly in the absence of ATP (Fig. 4B, *lanes 1–4 and 6–9*), in agreement with previously published results [25,29] on short 3' overhangs. The faint digestion pattern observed in reactions containing the 10 nt overhang substrate (Fig. 4B, *lanes 7–9*) was due to a low level of labeled single-stranded (unannealed) oligomer in this substrate preparation. This observed length dependence for exonuclease activity was related directly to overhang length and not nucleotide sequence, as under identical conditions WRN readily digests the comparable single-stranded oligomers ranging from 50 to 70 nt (Fig. 4B, *lanes 5, 10, 15, 20, 25*). Thus, the overhang length determines the ability of WRN to degrade such a structure in the absence of ATP, with a minimum of ≥ 20 nt needed to permit significant digestion at the 3' end. It is also noteworthy that, even on these long overhangs, the extent of inward degradation is very limited and did not increase significantly over time.

### Relationship between WRN exonuclease and ATPase/helicase activities on overhang substrates

Because of the profound impact of ATP on degradation of single-stranded oligomers, the effect of nucleotide cofactors on WRN degradation of our overhang substrates was also examined. Thus, WRN-mediated degradation of various length overhangs was directly compared in reactions without or with ATP or ATPγS; in addition, degradation by ATPase- and helicase-deficient WRN-K577M was examined in the presence of ATP (Fig. 5A). Importantly, ATP markedly stimulated both the initial level and the inward extent of degradation by wild type WRN on each overhang substrate. Most strikingly, the 5, 10, and 15 nt overhangs that were not significantly degraded in the absence of ATP are readily digested in its presence (Fig. 5A, *lanes 4, 9, and 14*). Degradation of the 20 and 25 nt overhangs was also most pronounced in the presence of ATP (Fig. 5A, *lanes 19 and 24*). The presence of ATPγS had an intermediate effect. Specifically, no degradation was observed on the 5 nt overhang (Fig. 5A, *lane 5*), but, on overhangs ≥ 10 nt, the initial level and extent of degradation in ATPγS were increased compared to reactions without ATP but not to the degree observed with ATP (Fig. 5A, *lanes 8–10, 13–15, 18–10, and 23–25*). Close examination of the digestion patterns indicates that the extent of inward degradation was limited in ATPγS as compared to ATP. Even incubation of up to 1 hr in ATPγS did not promote further inward degradation by WRN (data not shown), suggesting that this relative inhibition is due to physical and not kinetic constraints. Importantly, degradation patterns for reactions containing WRN-K577M (Fig. 5A, *lanes 2, 7, 12, 17, and 22*) were essentially identical to those for wild type WRN in the absence of ATP. When compared to the different digestion patterns observed in ATPγS, this indicates that the substitution of methionine for the conserved lysine in helicase motif I of WRN-K577M abrogates not only ATP hydrolysis but also ATP binding. Taken together, these results clearly demonstrate that ATP hydrolysis by the WRN ATPase/helicase domain dramatically and optimally stimulates degradation of 3' overhangs by the exonuclease domain.

A possible explanation for these results was that these overhang substrates were completely unwound by the ATPase-dependent helicase activity of WRN and the resulting single-stranded DNA (≥ 50 nt) was then degraded by its exonuclease function. To investigate this possibility, reactions containing WRN and the 10 nt 3' overhang substrate in the presence or absence of ATP were monitored simultaneously for degradation and unwinding over 10 min. In agreement with the results above, extensive stepwise degradation of this substrate by WRN in the presence of ATP was evident over time with more than 60% of the original substrate degraded by one nucleotide or more after only 2.5 min, while no significant degradation was observed in the absence of ATP (Fig. 5B, *top*); the faint pattern of exonuclease activity observed without ATP was due to digestion of a small amount of single-stranded 55-mer in this overhang substrate preparation. As expected, no unwinding was observed without ATP (Fig. 5B, *bottom*, *lanes 1–5*). Intriguingly, the analysis of unwinding in the presence of ATP (Fig. 5B, *bottom panel, lanes 6–10*) showed no significant increase in the amount of single-stranded species, either undegraded or degraded (faster migrating species than the 55 nt marker). At all time points, the amount of duplex form remained greater than 92%, indicating little or no complete unwinding of the substrate at these WRN concentrations. However, there was, over time, modestly faster migration of the duplex substrate, demonstrating that WRN exonuclease is acting on a structure retaining double-stranded character (Fig. 5B, *bottom panel, lanes 6–10*). When this analysis was performed with the same concentration of exonuclease-deficient WRN-E84A, no degradation or unwinding was observed (see additional file 1), confirming again that the observed exonuclease activity is inherent to WRN. Notably, much higher concentrations of both wild type WRN and WRN-E84A were needed to achieve significant unwinding of this substrate (data not shown). These results indicate that ATP hydrolysis can facilitate digestion of an overhang substrate without its complete unwinding, and suggest coordination between the ATPase/helicase and exonuclease domains of WRN to achieve degradation on accessible 3' overhangs.

Next, we examined whether the extent of ATP-stimulated degradation was limited on these 3' overhang substrates by monitoring WRN-mediated degradation of the 5, 10, and 15 nt overhangs over 60 min (Fig. 5C). This analysis demonstrated that, for all three substrates, degradation proceeds inward during the first 30 min but not much further thereafter. Close inspection and comparison of the digestion patterns revealed that, for each substrate, digestion occurred readily until the length of the overhang strand was shortened to 45–50 nt with weaker or slower degradation further inward. For some substrate molecules, the overhang strand was digested to approximately 25 nt without further inward degradation. This indicates that digestion was eventually limited by decreasing substrate length. This 25 nt/bp limit for inward digestion of these overhang substrates is similar to the minimum single-stranded oligomer length necessary to observe exonuclease activity, suggesting that a specific spatial factor (likely related to the molecular size of WRN) is operative in both situations.

Binding of WRN to these overhang substrates was also examined by EMSA. In these experiments, exonuclease-deficient WRN-E84A protein was used to prevent degradation of the labeled overhang strand. EMSA experiments were performed in ATPγS with substrates containing 5–25 nt overhangs, as detection of stable protein-DNA complexes was again optimal under these conditions (data not shown). The results of these experiments clearly indicated that WRN-E84A did bind to substrates with 3' overhangs (Fig. 5D). Although weak binding to the 5, 10, and 15 nt overhangs was detected at the highest WRN-E84A concentrations, significantly higher levels of binding were observed for the 20 and 25 nt overhangs (Fig. 5E). This suggests that, for 3' overhangs as well as perhaps other multi-stranded structures, a single-stranded region of ≥ 20 nt promotes WRN binding. Although this enhanced WRN binding affinity for longer overhangs may underlie the limited digestion of the 20 and 25 nt overhangs observed in ATPγS or the absence of ATP (see Fig. 4B), clearly the levels of exonuclease activity in the presence of ATP on shorter overhangs (Fig. 5A–C) do not directly reflect only binding stability as measured here. Importantly, our results suggest that optimal exonuclease activity is the result of an ATPase-mediated dynamic interaction of WRN with DNA substrates in which binding affinity is an important factor but not the only factor.

### Effect of other ribonucleotides on WRN exonuclease activity

**Figure 6:**
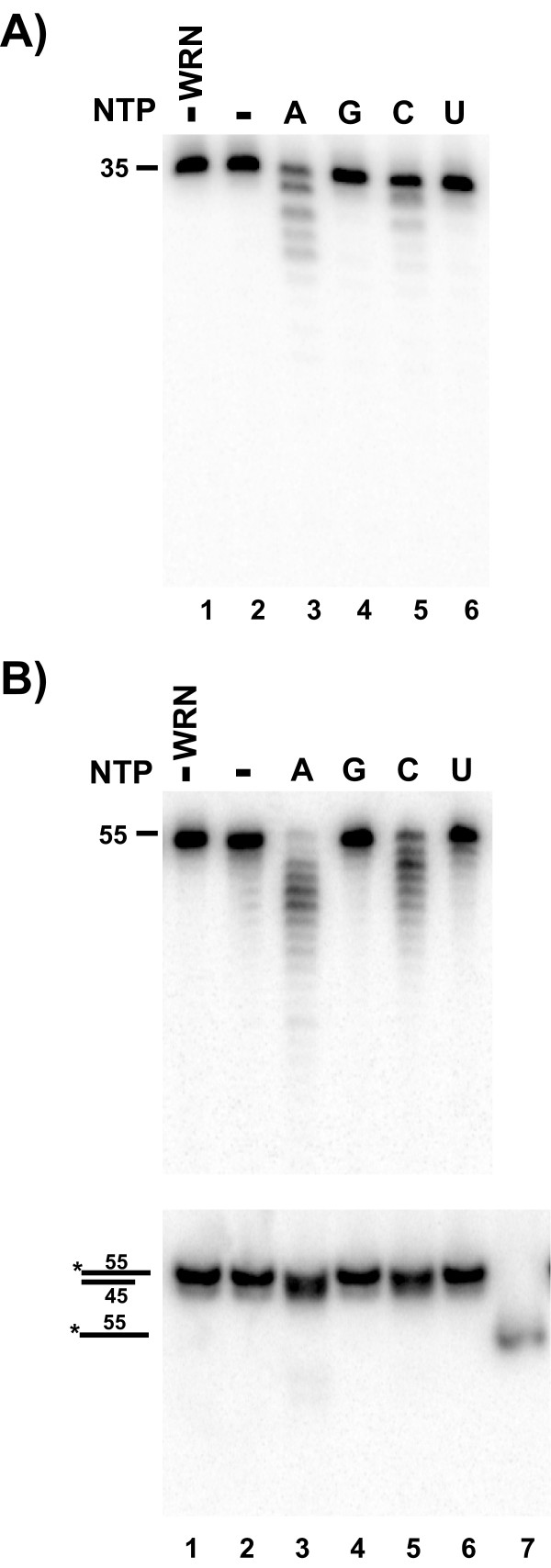
**Effect of various ribonucleotide cofactors on length-dependent WRN exonuclease activity. A) **Wild type WRN (2 nM) was incubated with labeled G35 (0.1 nM) for 10 min at 37°C without or with 1 mM ATP (A), GTP (G). CTP (C), or UTP (U) as indicated. DNA products were analyzed as described for the exonuclease assay in Methods. **B) **Wild type WRN (2.7 nM) was incubated with the 10 nt 3' overhang (labeled C55/G45) substrate (0.25 nM) for 10 min at 37°C without or with 1 mM ATP (A), GTP (G), CTP (C), or UTP (U). Reactions were analyzed in parallel for exonuclease (*top panel*) and unwinding (bottom panel) activities as described in Fig. 5B; heat-denatured substrate was used as a marker for single-stranded C55 (*lane 7, bottom panel*).

To determine whether this stimulatory effect on WRN exonuclease activity was specific to ATP, possible effects of other ribonucleotides were examined. In experiments with the 35-mer, again the stimulatory effect of ATP on WRN exonuclease activity was observed (Fig. 6A, *lanes 2 and 3*). Replacement of ATP with either GTP or UTP did not permit detectable degradation (Fig. 6A, *lanes 4 and 6*). However, the presence of CTP mediated a modest stimulation of WRN exonuclease activity (Fig. 6A, *lane 5*), although less than that observed with ATP. Similarly, significant degradation of the 10 nt overhang substrate was observed only when ATP or CTP was present, with more activity in ATP than CTP (Fig. 6B, *top*). In the parallel assessment of unwinding (Fig. 6B, *bottom*), no significant amount of single-stranded species is observed under any condition, in agreement with earlier results (Fig. 5B) that showed stimulation of exonuclease activity on this substrate in the presence of ATP without concomitant unwinding. Thus, CTP can stimulate WRN exonuclease activity in a manner similar to but somewhat less efficient than ATP. These results are consistent with an earlier report demonstrating that CTP (but not GTP or UTP) can substitute for ATP during unwinding of a short (20 bp) duplex [20].

### DNA binding and exonuclease activities of the isolated exonuclease domain of WRN

**Figure 7:**
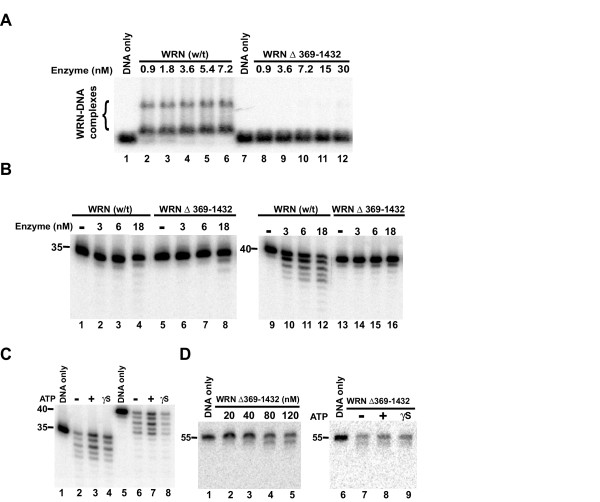
**DNA binding and exonuclease activities of the isolated exonuclease domain of WRN. A) **Wild type WRN or the exonuclease domain-only deletion mutant WRNΔ369-1432 (0.9, 3.6, 7.2, 15 or 30 nM each) was incubated for 5 min at 37°C with G45 (0.025 nM) in the presence of ATPγS (1 mM). Unbound DNA and DNA-protein complexes were separated using EMSA as described in Methods. **B) **Wild type WRN or WRNΔ369-1432 (3, 6 or 18 nM each) was incubated for 15 min at 37°C with single-stranded, radiolabeled G35 (0.1 nM, *left*) or G40 (0.1 nM, *right*) in the absence of ATP. **C) **Similarly, WRNΔ369-1432 (120 nM) was also incubated with either G35 or G40 (0.1 nM each) in the absence or presence of ATP or ATPγS (1 mM) for 15 min at 37°C. **D) **Standard exonuclease assays were performed on 10 nt 3' overhang substrate (labeled C55/G45, 0.5 nM) with WRNΔ369-1432 (20, 40, 80 or 120 nM) in 1 mM ATP for 10 min at 37°C (*left*). Similar reactions were performed with 10 nt overhang substrate (0.25 nM) and WRNΔ369-1432 (120 nM) in the absence or presence of ATP or ATPγS (1 mM) for10 min at 37°C (*right*). For *B-D*, exonuclease assays were performed as described in Methods. Lengths of the original substrates (in nt) are indicated at left of each panel.

The results above strongly suggest that dynamic interaction of the centrally located ATPase/helicase domain of WRN with particular DNA structures (demonstrated here on single-stranded DNA and 3' overhang substrates) has a positive effect on the N-terminal exonuclease function. If this were the case, the isolated WRN exonuclease domain would have significantly altered activities on these substrates. Therefore, DNA binding and exonuclease assays were performed using a deletion mutant (hereafter referred to as WRNΔ369-1432) containing only the N-terminal 368 amino acids that includes the entire exonuclease domain but essentially lacks the remainder of the protein. This truncated protein retains 3' → 5' exonuclease activity [24,30]. First, WRNΔ369-1432 and wild type WRN were directly compared for their ability to bind a 45-mer (Fig. 7A). As before, wild type WRN bound readily to this substrate under ATPγS conditions, with most of the DNA being shifted at the lowest concentration (0.9 nM) tested. In contrast, binding of WRNΔ369-1432 was only barely detectable at concentrations of 15 nM and above. As might be expected, the binding affinity of WRNΔ369-1432 (lacking the ATPase/helicase domain) for this oligomer was essentially identical whether the binding reaction contained ATPγS or ATP, or lacked nucleotide cofactor (data not shown). Thus, WRNΔ369-1432 has much weaker binding affinity for single-stranded DNA than wild type WRN.

Next, wild type WRN and WRNΔ369-1432 exonuclease activities were directly compared on both single-stranded and 3' overhang substrates. Since wild type WRN exonuclease activity in the absence of ATP was abruptly inhibited when oligomer length was ≤ 35 nt (see Fig. 1B), 35- and 40-mers were selected for analysis of the length and nucleotide dependence of WRNΔ369-1432. In agreement with the results above, WRN (at concentrations < 18 nM) removed several nucleotides from the 40-mer while digestion of the 35-mer was barely detectable (Fig. 7B, *compare lanes 1–4 with 9–12*). In contrast, no digestion was detected on either substrate using comparable WRNΔ369-1432 concentrations. Very modest digestion of both substrates was observed at a higher WRNΔ369-1432 concentration (18 nM), but with no significant difference in the amount or extent of degradation between the 35- and 40-mers (Fig. 7B, *lanes 5–8 and 13–16*). Wild type WRN, at this same concentration, still demonstrated a dramatic length dependence (Fig. 7B, *lanes 4 and 12*). With a much higher concentration (120 nM) of WRNΔ369-1432, significant digestion of both the 35 and 40 nt oligomers was achieved (Fig. 7C). However, there is still no discernable difference between the level and extent of digestion between the 35- and 40-mers. Furthermore, there was no significant effect of nucleotide cofactors on digestion patterns of these oligomers using WRNΔ369-1432 (Fig. 7C). We went on to test the ability of WRNΔ369-1432 to act on our 10 nt 3' overhang substrate that was highly degraded by wild type WRN in the presence of ATP but not in its absence (see Figs. 4B and 5A). In contrast to wild type WRN, the ability of WRNΔ369-1432 to digest this 10 nt overhang in the presence of ATP was low even at very high protein concentrations (Fig. 7D, *left*). As with single-stranded oligomers, WRNΔ369-1432 digestion of this overhang substrate was unaffected by nucleotide cofactors (Fig. 7D, *right*).

In summary, when directly compared to wild type WRN, the isolated exonuclease domain of WRN is much weaker in DNA binding and therefore its exonuclease activity on single-stranded oligomers and overhang substrates is significantly reduced, with neither property influenced by nucleotide cofactors. Moreover, the marked effect of oligomer length on wild type WRN exonuclease activity is not observed using the isolated exonuclease domain. Thus, our results suggest that the effects of oligomer and overhang length on the exonuclease activity of wild type WRN are the result of the orientation of the N-terminal exonuclease domain with respect to the remainder of the protein when bound to these substrates. Furthermore, our results demonstrating the marked stimulation of exonuclease activity by ATP hydrolysis specifically on oligomers and overhangs of limited length, suggests a dynamic relationship between the ATPase/helicase domain of WRN and DNA that results in accessibility of the 3' end of the substrate to the exonuclease domain and its subsequent digestion.

## Conclusion

Of the five human RecQ members, only WRN possesses an exonuclease function in addition to the requisite ATPase/helicase activity, implying that loss of both activities might be involved in eliciting the unique WS phenotype. In this study, the relationship of WRN 3' → 5' exonuclease activity to its ATPase/helicase function was illuminated using single-stranded and 3' overhang substrates. First, our results now establish that these types of DNA structures are authentic substrates for exonuclease degradation by WRN alone and reveal that the length of single-stranded regions in these substrates is critical in determining whether WRN can both initially attack and digest inward from their 3' ends. These findings indicate that WRN is a much more versatile exonuclease (and enzyme) than originally believed and significantly expand its potential *in vivo *structure specificity. Importantly, our results demonstrate that WRN-mediated digestion of these substrates is markedly enhanced by ATP binding and hydrolysis in a manner also influenced by DNA substrate length. Similar experiments using a deletion mutant containing only the N-terminal exonuclease domain show severely reduced DNA binding and exonuclease activities with no effect of ATP. Most importantly, our results indicate not only catalytic coordination but also a relative spatial orientation between the exonuclease and ATPase/helicase domains of WRN with respect to DNA substrate size and structure. Our results suggest rational models for the concerted, unidirectional 3' → 5' action of WRN's ATPase/helicase and exonuclease domains on DNA. These findings should help elucidate the physiological role of WRN in DNA metabolism.

Our analysis indicates that the lengths of both the overall substrate and any available single-stranded regions are critical factors in determining whether WRN acts as an efficient exonuclease. In the absence of ATP, WRN exonuclease activity cannot be detected on oligomers < 40 nt, in relative agreement with earlier reports using oligomers of ≤ 24 nt [25,29]. However, our results demonstrate that oligomers of ≥ 40 nt are substrates for WRN exonuclease, with the initial amount and extent of inward degradation from the 3' end increasing as oligomer length increases. Similarly, WRN can degrade a 3' overhang if the overhang length is ~15–20 nt or longer, while shorter overhangs are not degraded in the absence of ATP. These results showing the inability of WRN to digest overhangs of < 15 nt are similar to earlier reports using 3' overhangs of ≤ 13 nt [25,29]. Thus, WRN can indeed degrade single-stranded DNA and 3' overhangs, provided the length of each is sufficient. Importantly, ATP hydrolysis by WRN permits degradation of even shorter oligomers and short 3' overhangs. CTP also supports this length-dependent enhancement of WRN exonuclease, consistent with an earlier report demonstrating that CTP (but not other ribonucleotides) can substitute for ATP in unwinding of short duplexes [20]. In combination with the reality that longer substrates better represent the physiological structure of DNA, our findings emphasize that the length of DNA substrates should be strongly considered in the design and interpretation of *in vitro *experiments performed with WRN.

An enzyme's ability to act on a DNA substrate is obviously influenced by its binding affinity. Although WRN binds weakly to single-stranded oligomers of < 35 nt, it binds increasingly well as oligomer length increases from 35 to 80 nt. Similarly, WRN binds with significantly higher affinity to 3' overhangs of ≥ 20 nt than to shorter overhangs. Thus, limitations on WRN degradation of short oligomers or overhangs may partially be due to poor binding affinity. Importantly, the relative pattern of length-dependent binding of single-stranded oligomers by WRN is preserved regardless of the presence of nucleotide cofactors. We speculate that some restrictions on WRN activities with regard to substrates and their single-stranded regions reflect both the molecular size and DNA binding requirements of the active form of WRN. However, DNA binding is not the only factor involved in determining exonuclease activity, as WRN (in the absence of ATP) still only shows limited degradation of 40- and 45-mers even though it binds them reasonably well, and ATP hydrolysis allows significant degradation of shorter oligomers and overhangs that are weakly bound. WRN has separable exonuclease and ATPase/helicase domains, and thus its structure is, at least, bipartite. The dramatic effect of ATP on the length dependence of exonuclease activity suggests that the ATPase/helicase domain is involved in the dynamic interaction between WRN and the DNA substrate. Our EMSA results (see Fig. 2A) suggest that ATP hydrolysis makes WRN binding to DNA more unstable or transient than ATP binding (as exemplified under ATPγS conditions). These results with WRN are consistent with the mechanisms of most helicases – i.e., ATP binding promotes DNA binding while ATP hydrolysis drives translocation and unwinding [33,34]. ATP-dependent translocation of WRN on single-stranded DNA is supported by the demonstration that long single-stranded substrates promote a higher rate of ATP hydrolysis than short oligomers [35]. Thus, we conclude that, while absolute DNA binding affinity is certainly involved in mediating any potential WRN activity, the dynamic movement of WRN with respect to the DNA substrate is also a highly important factor in determining the ability of the exonuclease domain to initially attack and extensively degrade any available 3' end.

Our data with the isolated exonuclease domain (WRNΔ369-1432) further support the conclusions above. First, the weak DNA binding properties of WRNΔ369-1432 indicate that regions of WRN outside of the exonuclease domain are predominantly responsible for binding to single-stranded DNA and other DNA structures. This is consistent with earlier data showing that the helicase domain and C-terminal regions of WRN show significant DNA binding properties [36]. Compared to wild type WRN, the exonuclease activity of WRNΔ369-1432 is inefficient but relatively consistent in its ability to digest a 10 nt 3' overhang and single-stranded oligomers of various length. The relatively poor exonuclease activity is most likely due to the aforementioned weak DNA binding ability of WRNΔ369-1432 and perhaps a completely distributive mechanism of action. Despite this poor activity, this deletion mutant can carry out digestion of 10 nt 3' overhangs and 35 nt single-stranded oligomers that, in the absence of ATP, are quite refractory to digestion by wild type WRN. Thus, the effect of the length of single-stranded regions on digestion that is so evident with wild type WRN appears not to be a critical factor in determining the specificity of the exonuclease domain itself. Lastly, nucleotide cofactors have no effect on the exonuclease activity of WRNΔ369-1432, in stark contrast to their differential effects on wild type WRN on the same substrates. This is not surprising, as WRNΔ369-1432 lacks the region that binds and hydrolyzes ATP. However, this result eliminates the possibility that nucleotide cofactors have a non-specific effect on exonuclease activity and confirms that effects on exonuclease digestion by wild type WRN are indeed attributable to their interaction with its ATPase/helicase domain.

Our findings convincingly demonstrate that exonuclease activity of WRN can be markedly influenced by nucleotide cofactors that are bound and hydrolyzed by its ATPase/helicase domain. The positive effect of ATP hydrolysis only becomes evident when some aspect of substrate length is limiting. Specifically, the initial patterns of WRN exonuclease activity on an 80-mer are identical in the presence or absence of ATP, but degradation of a 45-mer is significantly better with ATP than without (see Fig. 1E). This experiment shows that binding or hydrolysis of ATP by the central ATPase/helicase domain does not alter the intrinsic exonuclease activity by a change in the overall WRN protein structure. Instead, it indicates that ATP hydrolysis changes the relationship between WRN and a DNA substrate of limited length such that a previously poor substrate for the exonuclease domain now becomes a much better substrate. This concept is further supported by our experiments that demonstrate a dramatic stimulatory effect of ATP hydrolysis on WRN-mediated degradation of substrates with 3' overhangs ranging between 5 and 25 nt (see Fig. 5A and 5B). Since the presence or absence of ATP has no effect on the rate of exonuclease digestion of a single-stranded oligomer of sufficient length, we conclude that ATP binding and hydrolysis influences the dynamic interaction of WRN with DNA in a manner dependent on substrate structure – i.e., in our experiments, the lengths of single-stranded substrates or 3' overhangs on the partial duplex substrates. This suggests that different modes of interaction of WRN with DNA are mediated by the presence or absence of ATP binding and hydrolysis that, in turn, have a significant effect on exonuclease activity. It appears that withholding ATP or allowing ATP binding but not hydrolysis mediate modes of interaction of WRN with substrates containing relatively short single-stranded regions that actually inhibit and/or limit digestion by the exonuclease domain. The effect of ATP binding without hydrolysis (exemplified by ATPγS conditions) on WRN degradation of 3' overhang substrates is intriguing. ATP binding permits significant but limited inward degradation of 10 and 15 nt overhangs that were not subject to WRN exonuclease activity without ATP but extensively degraded under conditions that allowed ATP hydrolysis; however, ATP binding does not allow degradation of the 5 nt overhang while ATP hydrolysis does (Fig. 5A). As mentioned above, formation of stable WRN-DNA complexes is promoted by ATP binding, but binding stability does not necessarily correlate to optimal exonuclease activity. Clearly, the positive effect of ATP binding is variable depending on the overhang length. We suggest that ATP binding (without hydrolysis) promotes a change in WRN conformation such that the exonuclease domain can attack the 3' ends of the 10 and 15 nt overhangs. However, this reorientation of WRN is not extensive enough to eliminate steric hindrance on the shorter 5 nt overhang and make it accessible to the exonuclease domain.

Since ATP hydrolysis both drives DNA translocation and unwinding by many enzymes with ATPase/helicase domains and optimally stimulates WRN exonuclease activity on limited length overhangs and oligomers, we suggest that the ATPase-mediated 3' → 5' translocation of WRN along DNA (with or without unwinding) underlies the associated stimulation of exonuclease activity. The most likely explanation for the restricted exonuclease activity on short overhangs or short oligomers under conditions where ATP hydrolysis is not allowed is that the binding modes of WRN actually obstruct access of the exonuclease domain to the 3' end. If the original length of the overhang or oligomer is sufficient, this type of obstruction and the associated inhibition of exonuclease activity only becomes apparent after some inward digestion when the length again becomes limiting. By this reasoning, ATP hydrolysis and movement of WRN along DNA in a 3' → 5' direction alleviates this obstruction, permitting access to the 3' end and digestion by the exonuclease domain. Although weak in DNA binding and thus relative exonuclease activity, the WRNΔ369-1432 mutant containing only the exonuclease domain is not obstructed by the DNA binding mode of the remainder of WRN. This also explains why, regardless of the presence or absence of nucleotide cofactor, the exonuclease activity of WRNΔ369-1432 is more or less independent of both 3' overhang and oligomer length. It is notable that other studies have observed that WRN exonuclease activity on several other DNA structures is promoted by ATP hydrolysis [10,25]. Our findings demonstrate that ATP hydrolysis stimulates WRN exonuclease in other structural contexts, but, most importantly, they specify the reason for this effect – i.e., the DNA substrate structure and length determine how WRN is oriented with respect to the particular 3' end to be digested. ATP hydrolysis and movement of the WRN helicase domain along DNA (with possible unwinding) alters this orientation and enhances exonuclease activity, particularly on substrates with single-stranded regions of limited length and possibly on *in vitro *substrates with short overall length. Perhaps more importantly for elucidation of the DNA metabolic role of WRN, our results imply that the activity of the exonuclease domain is directly connected to the function of its ATPase/helicase domain in moving WRN along DNA.

**Figure 8:**
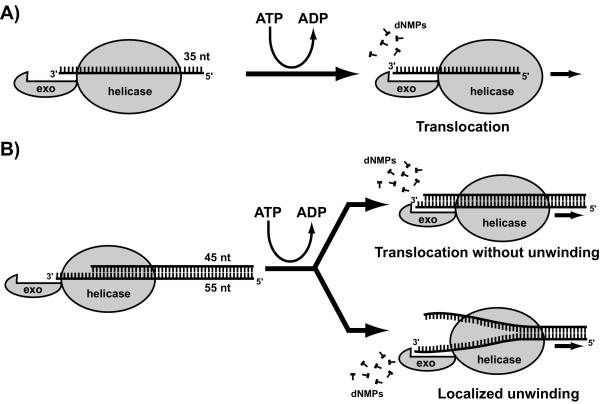
**Coordinated movement of WRN ATPase/helicase and exonuclease. **Models for the concerted 3' to 5' movement of the ATPase/helicase and exonuclease domains of WRN on single-stranded (*top*) and 3' overhang substrates (*bottom*). WRN is shown as a bipartite complex with distinct helicase and exonuclease domains. At left is the suggested binding mode of WRN on a short (35 nt) oligomer or short (10 nt) 3' overhang substrate in the absence of ATP. ATP hydrolysis allows movement of the helicase domain in a 3' to 5' direction along either single-stranded or duplex DNA, with movement of the tethered exonuclease domain and concomitant stepwise degradation of DNA (indicated by dNMPs) in the same direction. On the 3' overhang substrate, ATP hydrolysis by WRN putatively mediates either translocation along the duplex without unwinding (*upper pathway*) or localized unwinding of the duplex (*lower pathway*).

Earlier models for coordination of WRN helicase and exonuclease functions suggested that their limited substrate specificity might force them to act in opposite or converging directions – i.e., with the helicase domain binding to a single-stranded region (or other structure) and the exonuclease domain accessing a nearby 3' nick or end on the opposite strand [30,37]. Although such models may explain experimental observations on very short *in vitro *substrates, our results suggest more rational models for coordination between its ATPase, helicase and exonuclease activities (Fig. 8). In our models, WRN is depicted as a bipartite protein with the ATPase/helicase and exonuclease domains moving and working in tandem along the same DNA strand. Previous biochemical studies indicate that WRN's DNA binding affinity and specificity is primarily determined by the ATPase/helicase domain and C-terminal elements [36] and preferential for single-stranded regions or junctions between single- and double-stranded DNA with much lower affinity for duplex regions [22,30,37]. We suggest that, when ATP is not present, WRN binding is static and oriented such that the exonuclease domain cannot access the 3' end of either a short oligomer (Fig. 8, *top left*) or a short 3' overhang (*bottom left*). Thus, the inability of the WRN to digest these structures may be due to steric hindrance caused by the remainder of the protein. This notion is supported by DNase I footprinting analysis of a bubble-containing substrate, showing that WRN binds to and protects a 35–40 nt/bp region [30]. However, we propose that ATP hydrolysis mediates movement of the helicase domain along one strand of DNA in a 3' → 5' direction, thus bringing the exonuclease domain into proximity to the 3' end and allowing removal of mononucleotides with the same directionality. On single-stranded DNA (Fig. 8, *top*), ATP hydrolysis promotes translocation of the helicase domain and thus, in agreement with our data, improves both the extent of inward degradation and the processivity of the tethered WRN exonuclease domain. On 3' overhang substrates (Fig. 8, *bottom*), WRN binds at the junction between single- and double-stranded DNA; access to the 3' end and significant inward degradation by the exonuclease domain is mediated by the 3' → 5' movement of the helicase domain, with either translocation along the duplex without unwinding or with local unwinding of the duplex. These alternatives are consistent with our results indicating that ATP hydrolysis enhances WRN-mediated degradation of 3' overhangs without concomitant unwinding of the entire duplex region (Fig. 5B). Although complete unwinding of short duplexes *in vitro *can be achieved using relatively high WRN concentrations, we postulate that the primary function for the WRN ATPase/helicase domain is localized unwinding during DNA metabolism of long duplex regions.

In analogous terms, this model suggests that WRN might work like holding down the backspace function on a computer keyboard – i.e., movement is kept one-dimensional while code is removed stepwise in the same direction. Similarly, the function of WRN in DNA metabolism may be to remove a sizable stretch of one strand of DNA in a 3' → 5' direction. Although this model may bring to mind 3' → 5' proofreading by replicative polymerases, it differs in two important ways: 1) in DNA polymerase holoenzymes, the polymerizing and proofreading (3' → 5' exonuclease) domains/subunits act divergently, and 2) the predominant *in vivo *substrate for replicative proofreading is a mismatched 3' terminus. Based on our data, we anticipate that *in vivo *substrates might be 3' ends or nicks within structures that are either significantly resected or disassembled by WRN. In this regard, WRN exonuclease can act at nicks [29] and this activity is also optimally enhanced under conditions that permit ATP hydrolysis (Orren et al., unpublished results). This type of activity could be valuable for metabolic events necessitating significant degradation of one strand of DNA such as exonucleolytic processing during mismatch repair or non-homologous end-joining pathways, disruption of aberrant recombination intermediates, regression of replication forks (with degradation of daughter strand DNA), or processing of telomeric structures (either linear 3' overhangs or higher order structures). Notably, several of these possibilities have been suggested as functions for WRN, and would be consistent with the illegitimate recombination and/or cellular senescence phenotypes of WRN-deficient cells.

In summary, this investigation into WRN-mediated digestion of single-stranded DNA or 3' single-stranded overhangs has revealed a specific relationship between its ATPase/helicase and exonuclease domains. ATP hydrolysis (along with possible localized unwinding of duplex regions of overhang substrates) by WRN mediates optimal exonuclease activity on these structures, but the length of the single-stranded region also plays a prominent role. Our results indicate that the interaction of WRN's ATPase/helicase domain with DNA is a critical factor that regulates exonuclease activity. It appears that the spatial and dynamic relationship between the exonuclease and ATPase/helicase domains of WRN with respect to a particular DNA structure is crucial in determining the action of WRN on that structure certainly *in vitro *and most likely *in vivo*. Our results imply that these domains act coordinately in a unidirectional (3' → 5') manner during WRN function in DNA metabolism.

## Methods

### Enzymes

Wild type and mutant WRN proteins were overexpressed and purified to near homogeneity essentially as described previously [35], except that 0.1% Nonidet-P40 (NP40) was included in all chromatography buffers. The purity of a representative wild type WRN preparation is evidenced by SDS-PAGE followed by silver staining (see additional file 2). The WRN-E84A mutant has a glutamate to alanine substitution mutation in the N-terminal nuclease domain that abolishes its exonuclease activity but preserves its ATPase and helicase functions [20,24]. The WRN-K577M mutant has a lysine to methionine substitution in motif I of the ATPase/helicase domain that eliminates both ATP hydrolysis and DNA unwinding activity but retains 3' → 5' exonuclease activity [20,27,30]. The WRNΔ369-1432 deletion mutant contains only the N-terminal 368 amino acids of WRN but also retains 3' → 5' exonuclease activity [20,30]. WRN protein concentrations were determined by comparison with standards of known concentration by spectrophotometry using the Bradford assay and/or by SDS-PAGE. The wild type and mutant WRN proteins were stored at -80°C prior to use.

### DNA substrates

Oligonucleotides for assays and duplex substrate construction were obtained from Integrated DNA Technologies (Coralville, IA). Oligonucleotide sequences are listed in Table 1. Single-stranded oligomers G80, G45, G40, G35, G30, G24, C70, C65, C60, C55, and C50 were labeled at the 5' end with ^32^P-γ-ATP (3000 Ci/mmol) and polynucleotide kinase (PNK), 3' phosphatase-free (Roche Molecular Biochemicals) and unincorporated nucleotides were removed using standard procedures. For only DNA binding assays with single-stranded oligomers, G80, G45, G40, G35, G30, and G24 each contained a 3'-PO_4 _moiety. This 3'-PO4 group remains intact when labeled with the 3' phosphatase-free PNK and blocks WRN exonuclease activity (Orren and Machwe, unpublished results). To generate 45 bp substrates with 3' overhangs ranging from 5–25 nt in length, labeled C70, C65, C60, C55, or C50 was annealed with a twofold excess of unlabeled G45 (in this instance containing a 3'-PO_4 _moiety to prevent WRN exonuclease attack on the unlabeled strand). Labeled single-stranded oligomer and overhang substrate preparations were subjected to non-denaturing polyacrylamide (12%) gel electrophoresis (PAGE), and purified using a gel extraction kit (Qiagen). Substrates were stored at 4°C. Quantitation of labeled substrate concentration was achieved by determining, in a 24 hr annealing reaction, the amount of unlabeled complementary oligomer necessary to convert a labeled oligomer completely into duplex form and applying the specific activity of the isotope.

### Exonuclease and helicase assays

To reproducibly assess WRN exonuclease and helicase activities under various conditions, individual assays were repeated at least two times. Representative gels for each type of experiment are presented in the panels of each figure. Radiolabeled single-stranded or 3' overhang DNA substrates (0.05–0.25 nM) were incubated with wild-type WRN (1.2–10.8 nM), WRN-E84A (22 nM), WRNΔ369-1432 (3–120 nM) or WRN-K577M (3 nM) in WRN reaction buffer (40 mM Tris-HCl (pH 8.0), 4 mM MgCl_2_, 0.1 mg/ml bovine serum albumin, 0.1% NP40, and 5 mM dithiothreitol) for varying time intervals at 37°C. Where indicated, the reaction buffer also contained 1 mM ATP or ATPγS. In processivity experiments, similar reactions containing labeled G80 (0.25 nM) were pre-incubated with WRN for 5 min on ice then transferred to 37°C. After incubation at 37°C for 2.5 min, excess competitor (unlabeled G80) was added and samples were removed at the indicated times. The 0 and 2.5 min time points were removed just prior to transfer of the reactions to 37°C and addition of competitor DNA, respectively. For analysis of WRN exonuclease activity, these reactions were terminated by addition of equal volumes of formamide loading dye (95% formamide, 20 mM EDTA, 0.1% bromphenol blue and 0.1% xylene cyanol) and the DNA products were denatured at 90°C for 5 min and separated by denaturing 14% PAGE in 1× TBE (90 mM Tris-borate, pH 8.0, 2 mM EDTA) for 1 h at 50 W. The gels were dried and DNA products were visualized using a Storm 860 phosphorimaging system and ImageQuant software (Molecular Dynamics, Sunnyvale, CA). The stepwise shortening of the 5' labeled strand is indicative of the 3' → 5' exonuclease activity of WRN.

In some experiments with 3' overhang substrates, reactions were analyzed in parallel for helicase (unwinding) activity. For these reactions, aliquots were removed and terminated with the addition of one-sixth volume of helicase stop dye (30% glycerol, 50 mM EDTA, 0.9% SDS, 0.25% bromphenol blue and 0.25% xylene cyanol). The DNA samples were separated by non-denaturing 8% PAGE in 1× TBE for 2 h at 120 V. Gels were dried and DNA products visualized as above. For assessing unwinding activity, the band intensities (minus appropriate background) for the double- and single-stranded DNA species were determined for the WRN-treated and untreated reactions. Percentage of duplex DNA is calculated as the ratio (× 100) of double-stranded species to the total amount of DNA (single- and double-stranded) for each reaction. WRN-dependent unwinding is calculated as the ratio (× 100) of single-stranded DNA species to the total amount of DNA in enzyme-treated reactions, including subtraction of the low percentage of single-stranded DNA present in untreated controls.

### Electrophoretic mobility shift assays (EMSA)

Labeled substrates (25–250 pM), either oligomers (G80, G45, G40, G35, G30, G24) or 3' overhangs (C70/G45, C65/G45, C60/G45, C55/G45, C50/G45), were incubated for 15 min at 37°C with WRN (0.6–7.2 nM), WRNΔ369-1432 (0.9–30 nM) or WRN-E84A (0.6–7.2 nM) in WRN reaction buffer (10 μl) with or without ATP or ATPγS (1 mM). For formation of ternary Ab-WRN-DNA (supershifted) complexes, a WRN-specific polyclonal antibody (Novus Biologicals, 0.5 μl of 1:40 dilution) was added after the initial incubation period for an additional 5 min at 37°C. After addition of 2 μl of EMSA loading dye (0.25% bromphenol blue in 30% glycerol), samples were subjected to agarose (0.8 %) gel electrophoresis at 4°C. After gel drying, free DNA and DNA-protein complexes were visualized by phosphorimaging. For each reaction, the amounts of all protein-DNA complexes (as measured by the cumulative radioactivity migrating slower than the free DNA band) and free DNA were quantitated including subtraction of background; the percentage of DNA bound at specific WRN concentrations was calculated by dividing the amount of shifted DNA by the total amount of DNA in each reaction/lane.

## Abbreviations

ATPγS: adenosine 5'- [γ-thio]triphosphate, EMSA: electrophoretic mobility shift assays, NP-40: Nonidet P-40, PAGE: polyacrylamide gel electrophoresis, PNK: T4 polynucleotide kinase, SDS: sodium dodecyl sulfate, WS: Werner syndrome

## Authors' contributions

AM participated in the design of the experiments and performed specific biochemical assays, prepared materials for publication, and helped draft the manuscript. XL carried out biochemical assays and prepared DNA substrates and purified proteins. DKO conceived of the study, oversaw the design of all experiments, and participated in drafting the manuscript. Each author has read and approved the final version of the manuscript.

## Supplementary Material

Additional File 1 **Specificity of WRN exonuclease activity on a 3' overhang substrate **Data showing that the exonuclelease activity observed on 3' overhang substrates is inherent to WRN protein. Click here for file

Additional File 2 **Silver stain analysis of purified recombinant WRN protein **Purified WRN analyzed by SDS-PAGE followed by silver staining. Click here for file

## Figures and Tables

**Table 1:** Sequences^1 ^of oligonucleotides used

C50
GCTGATCAACCCTACATGTGTAGGTAACCCTAACCCTAACCCTAAGGACA
C55
GCTGATCAACCCTACATGTGTAGGTAACCCTAACCCTAACCCTAAGGACAACCCT
C60
GCTGATCAACCCTACATGTGTAGGTAACCCTAACCCTAACCCTAAGGACAACCC-TAGTGA
C65
GCTGATCAACCCTACATGTGTAGGTAACCCTAACCCTAACCCTAAGGACAACCCTAGTGAAGCTT
C70
GCTGATCAACCCTACATGTGTAGGTAACCCTAACCCTAACCCTAAGGACAACCCTAGTGAAGCTTGTAAC
G24^2^
CCTACACATGTAGGGTTGATCAGC
G30
GGGTTACCTACACATGTAGGGTTGATCAGC
G35
GGTTAGGGTTACCTACACATGTAGGGTTGATCAGC
G40
GTTAGGGTTAGGGTTACCTACACATGTAGGGTTGATCAGC
G45
TTAGGGTTAGGGTTAGGGTTACCTACACATGTAGGGTTGATCAGC
G80
CTTCACAGTCAGAGTCACAGTGTGCCGGGTTGTCCTTAGGGTTAGGGTTAGGGTTACCTACACATGTAGGGTTGATCAGC
^1^All sequences are depicted in 5' to 3' orientation. ^2^For EMSA on single-stranded G24, G30, G35, G40, G45. and G80, each oligomer included a 3'-PO_4 _moiety to block WRN exonuclease activity; overhang substrates were constructed with G45 with a 3' PO_4 _group, also to block potential WRN exonuclease activity on the unlabeled strand.
